# Malignant hypertension: current challenges, prevention strategies, and future perspectives

**DOI:** 10.3389/fcvm.2024.1409212

**Published:** 2024-12-24

**Authors:** Abate Wondesen Tsige, Siraye Genzeb Ayele

**Affiliations:** ^1^ School of Pharmacy, College of Health Sciences, Debre Berhan University, Debre Berhan, Ethiopia; ^2^ Department of Midwifery, College of Health Sciences, Addis Ababa University, Addis Ababa, Ethiopia

**Keywords:** malignant hypertension, risk factors, complication, treatment, prevention strategies

## Abstract

**Introduction:**

Based on office blood pressure (BP) values, hypertension is categorized into three stages: stage 1 (140–159/90–99 mmHg), stage 2 (160–179/100–109 mmHg), and stage 3 (≥180/≥110 mmHg). Malignant hypertension (MHT) is characterized by extreme BP elevation (systolic blood pressure above 200 mmHg and diastolic blood pressure above 130 mmHg) and acute microvascular damage affecting various organs, particularly the retinas, brain, and kidneys.

**Objectives:**

The pathogenesis, predisposing variables, therapy, and preventive strategies for MHT were examined in this review.

**Conclusions and recommendations:**

Malignant hypertension requires prompt and efficient treatment because it is the most severe kind of hypertension that affects target organs. At the same time, there are a number of alternatives available for treating MHT. The International Society of Hypertension 2020 and European Society of Cardiology/European Society of Hypertension 2018 recommendations suggest using labetalol and nicardipine as the first-line choice, with urapidil and nitroprusside serving as alternative medications. Elevated risk of MHT has been linked to many socio-demographic and genetic factors.

## 1 Introduction

According to the 2023 European Society of Hypertension (ESH) guideline, hypertension (HTN) is defined as a recurrent office systolic blood pressure (SBP) value of 140 mmHg and diastolic blood pressure (DBP) value of 90 mmHg. Based on office blood pressure (BP) values, HTN is categorized into three stages: stage 1 (140–159/90–99 mmHg), stage 2 (160–179/100–109 mmHg), and stage 3 (≥180/≥110 mmHg) (1).

Malignant hypertension (MHT) is characterized by extreme BP elevation (SBP above 200 mmHg and DBP above 130 mmHg) and acute microvascular damage affecting various organs, particularly the retinas, brain, and kidneys (2–4).

The 2018 European Society of Cardiology (ESC)/ESH guideline differentiates MHT from “hypertension urgencies and emergencies” through the severity of damage to the organs, which is referred to as hypertension-multi-organ damage (retinopathy, microangiopathy, disseminated intravascular coagulation, encephalopathy, abrupt heart failure, or acute deterioration in renal function). It is a medical emergency with a poor prognosis if left untreated (5).

MHT requires emergency medical attention to limit organ damage and other complications related to severely high blood pressure (6). Vascular injury is mainly caused by the loss of autoregulation of blood flow, which typically occurs in individuals with uncontrolled and chronic hypertension (3, 7, 8).

There is limited data on MHT so the diagnostic and therapeutic guidelines are primarily based on consensus. The different definitions of MHT in the guidelines are listed in Table 1.

**Table 1:** Agreements and disagreements regarding the definition of malignant hypertension in current guideline/consensus documents (3).

Guidelines	Definition	Agreement and disagreement
2023 ESH Guidelines (1)	This guideline does not define MHT but it does categorize HTN into three grades. Grade 3 HTN (≥180/≥110 BP) with the presence of CVD or CKD stage 4 or 5.	
BIHS position document (9)	Diastolic blood pressure has a value greater than 120 mmHg, and the Keith and Wagner classification needs concurrent symmetrical grade 3 (flame or dot-shaped hemorrhages, cotton wool spots, hard exudates, and microaneurysms) or grade 4 hypertensive retinopathy of the eyes (bilateral papilledema) to make a clinical diagnosis.	The authors agreed that more research is required in this area and that there is a lack of consensus statements regarding the exact rate of BP lowering.
ISH 2020 (10)	Severe symmetrical retinopathy with a large increase in arterial blood pressure (which is frequently >200/120 mmHg) with bleeding, cotton wool spots, and papilledema.	Although a significant increase in blood pressure is required, no particular threshold has been verified. MHT can be detected in this situation when significant retinopathy due to hypertension is present. This is agreed upon. It is debatable if it is necessary when there is heart, kidney, brain, or thrombotic microangiopathy damage, or when there is bilateral retinal involvement or papilledema. Severe hypertensive retinopathy may also be accompanied by isolated dry exudates, cotton wool patches, and hemorrhages.
NICE 2019 (11)	Blood pressure increases quickly to 180/120 mmHg or above (and typically >220/120 mmHg), together with symptoms of retinal bleeding and/or papilledema (optic nerve enlargement). Typically connected with new or newly developing major organ harm.	The committee concluded that more investigation was required. Neither published evidence nor pertinent clinical studies were found during the review process.
ESC/ESH 2018 (5)	Malignant hypertension is a hypertensive emergency characterized by the presence of severe BP elevation (usually >200/120 mmHg) and advanced retinopathy, defined as the bilateral presence of flame-shaped hemorrhages, cotton wool spots, or papilledema.	The authors claimed that when estimating the severity of organ injury, the degree and magnitude of BP increases can be as significant as absolute BP level.
European Consensus 2018 (8)	The presence of severe retinopathy (defined by the bilateral presence of flame-shaped hemorrhages, cotton wool patches, or papilledema), acute renal failure, thrombotic microangiopathy, and acute hypertensive microangiopathy could be an alternative term with elevated blood pressure (typically 200/120 mmHg).	In support of a larger definition, it is argued that retinal lesions may not always be present in individuals who have had acute microvascular damage to the kidney, heart, or brain. This is due to evidence gaps and the disease's pathophysiology.
AHA 2017 (6)	Not brought up in the discussion.	The absence of malignant hypertension in the section on emergencies with hypertension reflects how the medical community overlooked MHT.
AHA 2024 Scientific Statement (12)	Does not define MHT as a distinct category in hypertension emergencies.	
2024 ESC Guidelines (13)	MHT is a hypertensive emergency characterized by extreme BP elevations and acute microvascular damage affecting various organs, particularly the retinas, brain, and kidneys.	The authors recommended safe BP reduction to prevent further complications.

BIHS, British and Irish Hypertension Society; AHA, American Heart Association.

## 2 Epidemiology of malignant hypertension

Although the prevalence and incidence data for MHT are scarce, a study conducted in Amsterdam (Netherlands) and Birmingham (United Kingdom) revealed an incident rate of 2/100,000 new cases per year, with up to fourfold higher rates among patients of Black African/Afro-Caribbean ethnicities (14). In the Afro-American population, 7.3/100,000 new cases per year were reported. A worse disease prognosis and greater disease predisposition were also observed (15).

## 3 Predisposing factors of malignant hypertension

Malignant hypertension mainly occurs in individuals with a history of uncontrolled high blood pressure and those who missed or discontinued their antihypertensive medications (16, 17). In addition, patients with stroke, structural heart disease, thyroid disorders, brain bleeding, renal artery disease, traumatic brain injury, Conn's syndrome, Cushing's syndrome, pheochromocytoma, and substance and medication withdrawal may experience MHT (17). In addition, pregnancy is another known precipitating factor of MHT (3, 18).

The study conducted in Birmingham included 460 patients diagnosed with MHT from 1958 to May 2016 and indicated that the study participants’ ethnic differences, advanced age, prior use of antihypertensive medication, duration of hypertension, and presence of proteinuria were strong predictors of MHT outcomes (19). There are more pronounced abnormalities in macrovascular and microvascular function in patients with MHT (which seem to be both endothelium-dependent and endothelium-independent) compared with patients with hypertension and healthy controls (20).

### 3.1 Socio-demographic factors

According to a study conducted on the Birmingham registry of patients with MHT, white Caucasian patients were more likely than African-Caribbean and South Asian patients to experience papilledema-related ocular problems (19). A study in Nigeria indicated patients with MHT had stressful lifestyles, were members of lower socio-economic groups, were older, had higher body mass indexes (BMI), had high BP, and had shorter in-time diagnoses of MHT (21).

Patients who had higher serum creatinine levels, who were current smokers, and who had no medical insurance had a significant association with MHT complications (22). A study conducted by Hertz et al. using the National Health and Nutrition Examination Survey (NHANES) 1999–2002 data indicated higher prevalence rates of hypertension in Black people compared to white people and a growing disparity in BP control among those treated using pharmacological agents (23).

### 3.2 Genetic factors

According to a study conducted using the Bordeaux, Birmingham, and Amsterdam MHT registries, ethnic minorities had a higher risk of MHT (15). Black patients had a higher incidence and more complications than white patients (22). In the USA, non-Hispanic Black patients had poor BP control compared to non-Hispanic white patients, and a higher prevalence of HTN was observed among non-Hispanic Black patients (24, 25).

Kalinowski et al. reported that Black patients’ endothelial cells had an increased release of both oxygen-free radicals and peroxynitrite and a reduced release of biologically active nitric oxide (NO). The study implied that there are differences in vascular function among patients of different races (26).

Another factor contributing to racial/ethnic pathophysiological differences in the mechanisms of HTN was BMI. The BMI of non-Hispanic Black patients was higher compared to non-Hispanic white patients and Chinese and Asian patients, resulting in a high prevalence of HTN (27).

Seven single-nucleotide polymorphisms (G589S, R597H, T594M, R624C, G442V, E632G, and V434M) were identified in the β subunit of the epithelial sodium channel (ENaC) (*SCNN1β* gene). The threonine-to-methionine point one mutation has only shown an association with HTN at position 594 (T594M). Persons of African origin have approximately 6% of the T594M allele, which is associated with elevated HTN risk in the African-American population, but this has not been found in any white patients (28).

## 4 Pathophysiology of malignant hypertension

There are various factors associated with the pathogenesis of MHT, although the underlying mechanism leading to MHT is not fully understood. Fibrinoid arteriolar necrosis in vascular tissue beds is the hallmark pathophysiological marker of MHT (9). Appropriate tissue blood perfusion is maintained by the auto-regulatory arterial and arteriolar processes which prevent the increase in pressure from being transmitted to the smaller, more distal vessels. During severe HTN, this autoregulation eventually fails and the vascular wall of arterioles and capillaries becomes damaged due to the rise in BP. The vascular endothelium then allows plasma constituents (including fibrinoid material) to enter the vascular wall, thereby narrowing or obliterating the vascular lumen (29).

**Figure 1:**
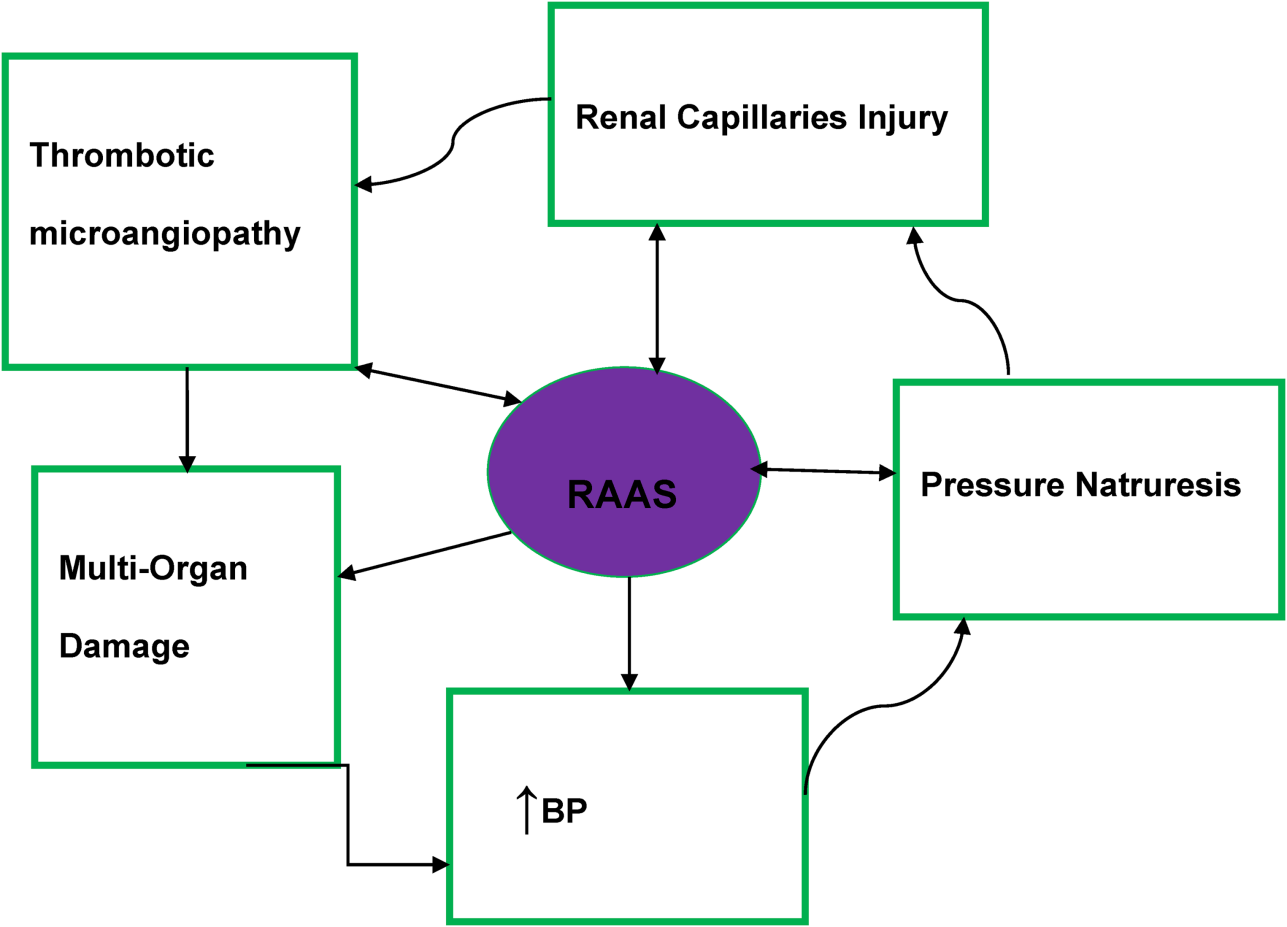
Malignant hypertension pathophysiology diagram. RAAS, renin–angiotensin–aldosterone system; BP, blood pressure.

Macrovascular and microvascular endothelium dysfunction is one of the abnormalities found in MHT patients (20). Thrombotic microangiopathies have resulted from the activation of the renin–angiotensin–aldosterone system (RAAS) and impairment of the intravascular prothrombotic state (agglutination/coagulation) along with excessive endothelial injury (20, 30) (Figure 1).

Significant activation of the RAAS is evident in patients with MHT and may also contribute to the development of fibrinoid necrosis (31). In patients with MHT, there is evidence that irrespective of the impact of BP elevation on plasma renin activity, angiotensin 2-dependent aldosterone secretion was more profound than in patients with severe HTN (32, 33).

The basic pathophysiology of MHT is summarized in Figure 1.

### 4.1 Symptoms of malignant hypertension

In many cases, there are no symptoms associated with high blood pressure. However, this is not the case with MHT (16). During the MHT phase, a visual disturbance is one of the MHT symptoms. Retinopathy secondary to MHT is divided into grades A and B. Grade A retinopathy includes arteriolar narrowing and focal constriction of the retina and it corresponds to non-MHT changes. Grade B retinopathy corresponding to MHT includes linear flame-shaped hemorrhages, exudates, and/or cotton wool spot changes with or without papilledema (34).

Furthermore, sudden onset of headache, nausea, vomiting, visual disturbances, and visual field loss [in some cases, the symptoms depend on the damaged organ (e.g., brain) and include restlessness, confusion, seizures, and coma] are the signs and symptoms of MHT encephalopathy (35, 36).

MHT also results from acute renal failure (37). Malignant hypertensive patients have been shown to have severe renal dysfunction and a good cardiovascular risk profile compared to patients with controlled HTN (38). A long follow-up period and close BP control are required to preserve renal function (32, 39).

Clinically, thrombotic microangiopathy (TMA) is defined as microangiopathic hemolytic anemia, thrombocytopenia, and increased serum lactate dehydrogenase (LDH), all of which are frequently seen in patients with MHT (30).

Pulmonary edema has also been associated with MHT (38).

## 5 Complications of malignant hypertension

Macrovascular and microvascular function abnormalities are one of the major complications of MHT (20, 40). Malignant hypertension can also lead to stroke and encephalopathy (20, 41), heart damage (angina, heart attack, and arrhythmia), kidney failure, vision damage (permanent blindness), and pulmonary edema (16, 17, 20, 40, 42, 43) (Table 2).

**Table 2:** Target organs damaged by MHT.

Target organs	Complication due to MHT
Heart	Heart attack, aortic dissection, chest pain (angina), and arrhythmias (irregular heartbeat)
Brain	Reversible encephalopathy syndrome
Kidneys	Proteinuria
Eyes	Retinopathy (serous retinal detachment)
Blood vessels	Fibrinoid necrosis

A single-center retrospective study conducted by Mishima et al. indicated that patients with MHT had a wide range of distribution patterns in the brainstem and 60% of the patients had posterior reversible encephalopathy syndrome (PRES) (44) (Table 2).

## 6 Prognostic factors of malignant hypertension

The prognosis is typically favorable for those who obtain an early diagnosis and suitable antihypertensive medication. Due to the disease's tendency to advance quickly and produce irreversible end organ damage, the interval between diagnosis and therapy is crucial (3). The death rate for untreated MHT is 80% within 2 years. MHT can be fatal even with treatment; one study found that across 25 US hospitals, the hospital death rate was close to 7% and the readmission rate was 37% within 90 days (15).

## 7 Assessment of malignant hypertension

### 7.1 Diagnosis of malignant hypertension

In many patients, the initial diagnosis of MHT is frequently missed which results in the diagnosis only being established when target organ damage occurs (14).

Some evidence suggests that a diagnosis of MHT can be made when a patient presents with acutely and quickly raised BP in cardiovascular disease (CVD) and chronic kidney disease (CKD) stages 4 and 5, respectively (1).To differentiate between a hypertensive urgency, emergency, or MHT, healthcare providers must perform a comprehensive assessment. A patient’s symptoms will determine the kinds of investigation tests they undergo, which could include an electrocardiogram (EKG), a chest x-ray, an eye exam to check for signs of bleeding or damage to the eyes, a neurological exam to assess brain function, a physical examination, a toxicology serum drug level study, a urinalysis, liver function tests, and renal function tests (17).

Malignant hypertension is diagnosed clinically when there is evidence of organ damage from a fundoscopic eye examination, a chest x-ray, urine analysis, blood tests, and high blood pressure (taken using both arms) at or above 180/120 mmHg (1, 2, 45).

In addition, one of several possible presentations is also hypertension-mediated multi-organ damage (2, 5). Typically, concurrent bilateral grade 3 (flame or dot-shaped hemorrhages, cotton wool spots, hard exudates, and microaneurysms) or grade 4 hypertensive retinopathy (bilateral papilledema), as defined by Keith and Wagner's classification, is required for a clinical diagnosis of MHT (9).

## 8 Malignant hypertension treatment and prevention methods

There are limited therapeutic options specifically indicated for MHT (3, 46), and its treatment depends on expert decision-making (11). According to the National Institute for Health and Care Excellence (NICE) guideline, there is no consensus on the BP targets, BP reduction frequency, or type of drugs to administer to patients when treating MHT. However, the goal of therapy for MHT is to rapidly lower the mean arterial pressure by approximately 10%–15% in the first hour, and by no more than 25% compared with baseline by the end of the first day of treatment (47) (Table 3).

**Table 3:** Agreement and disagreement in latest hypertension guidelines regarding BP reduction rate and BP target in malignant hypertension.

Guidelines	BP reduction rate and BP goal	Drug choice	Agreement and disagreement
2023 ESH (1)	The target blood pressure range for adults is below 130/80 mmHg for those 18–64 years old, below 140/80 mmHg for those 65–79 years old, and 140–150/80 mmHg for those 80 years and above.	Labetalol and nicardipine are the first-line treatment. Nitroprusside and urapidil are the alternatives.	There seems to be agreement with the ESC/ESH 2018 and ISH 2020 guidelines. There is disagreement with the AHA/ACC 2017 and NICE 2019 guidelines.
ISH 2020 (10)	Reduce mean arterial pressure (MAP) by 20%–25% over several hours.	Labetalol and nicardipine are the first-line treatment while nitroprusside and urapidil are the alternatives.	There seems to be agreement with the ESC/ESH 2018 and 2023 ESH guidelines. There seems to be disagreement with the AHA/ACC 2017 and NICE 2019 guidelines.
NICE 2019 (11)	There are no BP targets, BP reduction rate, or specific medications for patients with MHT.	No specific antihypertension medications.	It seems to be in disagreement with the ESC/ESH 2018, ISH 2020, and 2023 ESH guidelines. There seems to be agreement with the AHA/ACC 2017 guideline.
ESC/ESH 2018 (5)	Reduce MAP by 20%–25% over several hours.	Labetalol and nicardipine are the first-line treatment. Nitroprusside and urapidil are the alternatives.	There seems to be agreement with the ISH 2020 and 2023 ESH guidelines. It appears that the AHA/ACC 2017 and NICE 2019 guidelines disagree with this guideline.
AHA/ACC 2017 (6)	Not clearly stated.	No specific antihypertension medications.	There seems to be disagreement with the ESC/ESH 2018, ISH 2020, 2023 ESH, and BIHS guidelines. However, there seems to be agreement with the NICE 2019 guideline.
BIHS position document (9)	For uncomplicated MHT, target BP <135/85 mmHg at home) within days to weeks (6–12 weeks). For complicated MHT, does not necessarily favor certain rates of BP lowering.	For uncomplicated MHT (eye changes only), 25 mg oral atenolol, 5 mg oral amlodipine/30 mg long-acting oral nifedipine. For complicated MHT, no specific antihypertension medications.	There is no full agreement with ESC/ESH 2018, ISH 2020, and 2023 ESH guidelines. There seems to be disagreement with the AHA/ACC 2017 and NICE 2019 guidelines.

AHA, American Heart Association; ACC, American Collage of Cardiology; BIHS, British and Irish Hypertension Society.

According to the British and Irish Hypertension Society regarding the speed of BP reduction and BP targets in uncomplicated MHT (eye changes alone), BP is to be lowered within days, aiming for a gradual reduction to reach the target BP within weeks. For example, within 24 h, BP should be lowered to <200/120 mmHg, within a week to <160/100 mmHg, and then to <140/90 mmHg within 6–12 weeks (9) (Table 3).

The latest hypertension guidelines provide a BP goal and the rate of BP reduction during treatment. Large and sudden reductions in BP must be avoided in patients with MHT as this can exacerbate ischemic lesions. The gradual increase in BP control allowed gradual healing of necrotizing vascular lesions (8) (Table 3).

Since MHT constitutes a hypertensive emergency, the ESC position document on the management of hypertensive emergencies advises using intravenous BP-lowering medicines in patients with acute presentations. Because there are no randomized controlled trials on various treatment approaches, the position paper does recognize that any recommendations are based on clinical experience and consensus (8, 42) (Table 3).

Parenteral antihypertensive drugs are used for the treatment of MHT (46, 48–51) (Table 4).

**Table 4:** Parenteral antihypertensive drugs used for the treatment of MHT.

Drugs	Dosage and administration
Clevidipine	Use 1–2 mg/h in an IV infusion with rapid titration to 4–6 mg/h and a maximum dose of 16 mg/h or less. Its duration of action is 5–15 min.
Enalaprilat	Administer 1.25–5 mg QID IV and the duration of action is approximately 6 h to more than 12 h.
Fenoldopam	Administer 0.1 μg/kg min in an IV with a maximum dose of 1.6 μg/kg/min. It has a 30–60 min duration.
Nicardipine	Uses 5–15 mg per hour in an IV infusion and the maximum dose we use is 30 mg/h. The duration of action is approximately 1.5 h to more than or equal to 4 h.
Nitroglycerin	Administer 5–100 μg/min in an IV infusion. It has a 5–10 min duration of action.
Nitroprusside	Administer 0.25–10 μg/kg per minute in an IV infusion. The cyanide toxicity is minimized by administering a short infusion and not exceeding 2 μg/kg/min. Its duration of action is 1–10 min. To avoid cyanide toxicity, patients who receive higher doses (i.e., >500 μg/kg at a rate exceeding 2 μg/kg/min) should receive a sodium thiosulfate infusion.
Esmolol	Use a 250–500 μg/kg loading dose over 1 min and then initiate an IV infusion at 25–50 μg/kg/min, using a maximum dose of 300 μg/kg min. Its duration of action is 10–30 min.
Labetalol	Administer a bolus dose of 20 mg followed by a 20–80 mg IV infusion every 10 min (maximum 300 mg). Its duration of action is 2–4 h.
Metoprolol	Administer 1.25–5 mg followed by a 2.5–15 mg IV infusion every 3–6 h. The duration of action is 5–8 h
Urapidil	Administer 10–50 mg with a slow IV injection every 5 min based on the patient's BP record.

Once the target BP range is achieved in patients with MHT, oral antihypertensive treatment is progressively introduced at the clinician's discretion (46, 50) (Table 4).

**Figure 2:**
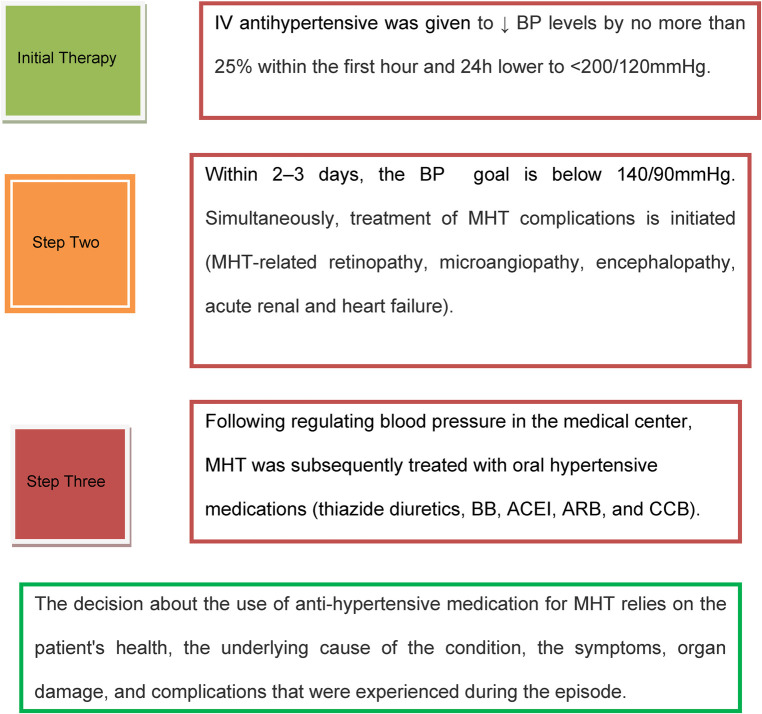
Malignant hypertension treatment protocol. IV, intravenous; MHT, malignant hypertension; BP, blood pressure; BB, beta-blocker; ACEI, angiotensin-converting enzyme inhibitor; ARB, angiotensin receptor blocker; CCB, calcium channel blocker.

Based on the reviewed works in the literature above and using the latest hypertension guidelines, the current treatment protocol for MHT is depicted in Figure 2 (5, 8, 46, 52, 53). A retrospective cohort study conducted by Endo et al. reported that in individuals experiencing hypertensive emergencies, early administration of renin-angiotensin system (RAS) inhibitors aids in the recovery of renal function following an abrupt decrease in estimated glomerular filtration rate (eGFR). In addition, compared to calcium channel blockers (CCB), renin-angiotensin system inhibitor (RASi) has a stronger positive impact on 2-year renal survival (54).

Non-pharmacological prevention modalities can also prevent MHT. Dr. Walter Kempner began using “The Rice Diet,” named thus because the patients consumed a bowl of white rice with every meal, to treat patients with kidney disease and MHT in 1934 while he was a physician at Duke Hospital. At the time, there was no other treatment for these conditions. Dr. Kempner realized that the best way to avoid and treat these disorders would be to follow a diet low in added salt and fat (55).

Therefore, it is advisable that patients with MHT frequently check their blood pressure, take their medicine as directed, eat a nutritious diet low in saturated fat and salt, stop smoking if they already do, and maintain a healthy weight (16).

Clinically, a significant risk factor for MHT is the self-discontinuation of antihypertensive medication by patients who have previously experienced MHT. Thus, long-term continuous follow-up is very important for prevention of MHT.

## 9 Conclusion and future perspectives

Malignant hypertension requires prompt and efficient treatment because it is the most severe kind of hypertension that affects target organs. There are a number of alternatives available for treating MHT, with the International Society of Hypertension (ISH) 2020 and ESC/ESH 2018 recommendations suggesting the use of labetalol and nicardipine as the first-line choice, with urapidil and nitroprusside serving as alternative medications.

An elevated risk of MHT has been linked to many socio-demographic factors and genetic factors. The international scientific community and governmental and non-governmental organizations, particularly those involved in hypertension research, should examine the new international MHT studies and conduct additional MHT research.
